# Dichlorido(dimethyl sulfoxide-κ*S*)(η^6^-mesitylene)ruthenium(II)

**DOI:** 10.1107/S160053681100314X

**Published:** 2011-02-12

**Authors:** Benjamin Oelkers, Lars Hendrik Finger, Jörg Sundermeyer

**Affiliations:** a Philipps-Universität Marburg, Fachbereich Chemie, Hans-Meerwein-Strasse, 35032 Marburg, Germany

## Abstract

The title compound, [RuCl_2_(C_9_H_12_)(C_2_H_6_OS)], features a planar [maximum deviation = 0.0075 (17) Å] η^6^-bound mesitylene ligand and a dimethyl sulfoxide ligand coordinated *via* the S atom. The overall complex geometry about the Ru(II) atom is best described as a piano-stool configuration.

## Related literature

For similar complexes of the type [RuCl_2_(DMSO)(arene)], see: Ogata *et al.* (1970 ▶) (arene = benzene); Chandra *et al.* (2002 ▶) (arene = *p*-cymene); Beasley *et al.* (1993 ▶) (arene = 1,4,9,10-tetra­hydro­anthracene); Haquette *et al.* (2008 ▶) (arene = 9,10-dihydro­anthracene); Sadler *et al.* (2005 ▶) (arene = 2-chloro-*N*-(2-phenyl­eth­yl)acetamide).
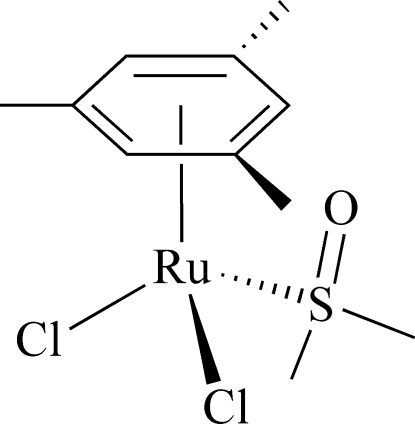


## Experimental

### 

#### Crystal data

[RuCl_2_(C_9_H_12_)(C_2_H_6_OS)]
*M* *_r_* = 370.28
Monoclinic, 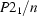

*a* = 8.1184 (4) Å
*b* = 22.9372 (13) Å
*c* = 8.3417 (4) Å
β = 116.443 (3)°
*V* = 1390.82 (12) Å^3^
*Z* = 4
Mo *K*α radiation
μ = 1.64 mm^−1^
*T* = 100 K
0.24 × 0.15 × 0.09 mm

#### Data collection

Stoe IPDS 2T diffractometer
Absorption correction: multi-scan (Blessing, 1995 ▶) *T*_min_ = 0.630, *T*_max_ = 0.994
9737 measured reflections
2935 independent reflections
2753 reflections with *I* > 2σ(*I*)
*R*_int_ = 0.051

#### Refinement

*R*[*F* ^2^ > 2σ(*F* ^2^)] = 0.017
*wR*(*F* ^2^) = 0.044
*S* = 1.04
2935 reflections
150 parameters
H-atom parameters constrained
Δρ_max_ = 0.42 e Å^−3^
Δρ_min_ = −0.71 e Å^−3^

### 

Data collection: *X-AREA* (Stoe & Cie, 2001 ▶); cell refinement: *X-AREA*; data reduction: *X-RED* (Stoe & Cie, 2001 ▶); program(s) used to solve structure: *SIR92* (Altomare *et al.*, 1993 ▶); program(s) used to refine structure: *SHELXL97* (Sheldrick, 2008 ▶); molecular graphics: *DIAMOND* (Brandenburg, 2007 ▶); software used to prepare material for publication: *WinGX* (Farrugia, 1999 ▶).

## Supplementary Material

Crystal structure: contains datablocks I, global. DOI: 10.1107/S160053681100314X/kj2168sup1.cif

Structure factors: contains datablocks I. DOI: 10.1107/S160053681100314X/kj2168Isup2.hkl

Additional supplementary materials:  crystallographic information; 3D view; checkCIF report

## Figures and Tables

**Table:** 

C1—Ru1	2.2209 (15)
C2—Ru1	2.2162 (15)
C3—Ru1	2.2219 (16)
C4—Ru1	2.2097 (16)
C5—Ru1	2.2160 (17)
C6—Ru1	2.1978 (15)
S1—Ru1	2.3399 (4)
Cl1—Ru1	2.4097 (4)
Cl2—Ru1	2.3963 (4)

**Table:** 

S1—Ru1—Cl2	85.030 (14)
S1—Ru1—Cl1	84.567 (14)
Cl2—Ru1—Cl1	88.636 (15)

**Fig. 1.**
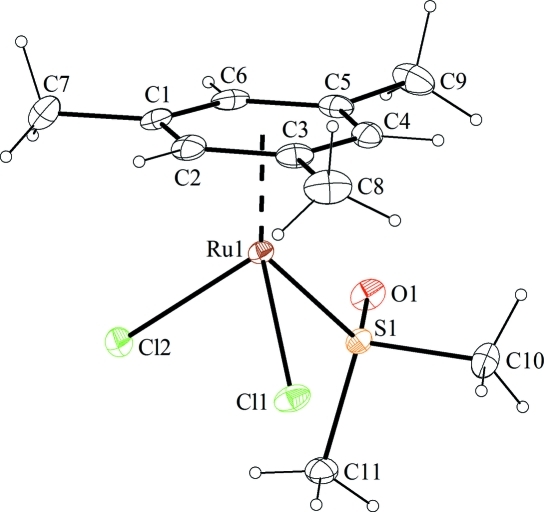
Molecular structure of the title compound. Displacement ellipsoids are shown for 50% probability.

## supplementary crystallographic information

### Comment

Complexes of the type [{RuCl_2_(arene)}_2_] are valuable starting materials for the preparation of ruthenium(II) complexes because they allow facile ligand substitution. During our investigations concerning the arene substitution behaviour of such complexes, we found that [{RuCl_2_(C_6_H_3_Me_3_)}_2_] readily reacts with dimethylsulfoxide, yielding the monomeric title compound [RuCl_2_(DMSO)(C_6_H_3_Me_3_)]. Similar reactivity has been reported for the corresponding benzene and *p*-cymene complexes (Ogata *et al.*, 1970; Chandra *et al.*, 2002).

The overall complex geometry of the title compound is best described as a piano-stool configuration. Another possible description is that of an octahedral d^6^ low-spin complex with the arene ligand occupying three *fac*-oriented coordination sites. The angles between the monodentate chloro and DMSO ligands are thus close to 90° (Table 1). The planar, η^6^-bound mesitylene ligand shows almost equal Ru–C distances of 2.1978 (15) to 2.2219 (16) Å. Dimethylsulfoxide is coordinated *via* sulfur as usual for complexes without sufficient steric bulk to force *O*-coordination. All numerical parameters concerning the molecular geometry are similar to those observed for the corresponding *p*-cymene complex (Chandra *et al.*, 2002).

### Experimental

[{RuCl_2_(C_6_H_3_Me_3_)}_2_] (175 mg, 0.30 mmol) was dissolved in DMSO (12 ml). The solution was heated to 100 °C for 45 min and a small amount of ruthenium black was removed by filtration. The dark red solution was concentrated *in vacuo* until the formation of crystals was observed. The product was isolated by filtration and dried *in vacuo*. A second crop of material was obtained from the mother liquor by layering with toluene. Yield: 150 mg (68%) of red crystals. Crystals suitable for X-ray diffraction were obtained by slow evaporation of a saturated DMSO solution.

### Refinement

Hydrogen atoms were placed on idealized positions and refined using a riding model with *U*_iso_(H) = 1.2 × *U*_eq_(C) (1.5 for methyl groups) and C–H bond lengths of 0.95 Å for aromatic protons and 0.98 Å for methyl groups.

Reflexes 0 1 1 and -1 1 1 were omitted from the refinement because they were effected by the diffractometer's beamstop. Reflex -5 0 3 was also omitted because of its exceptionally large deviation from the calculated intensity.

### Crystal data

**Table:**

[RuCl_2_(C_9_H_12_)(C_2_H_6_OS)]	*F*(000) = 744
*M**_r_* = 370.28	*D*_x_ = 1.768 Mg m^−^^3^
Monoclinic, *P*2_1_/*n*	Mo *K*α radiation, λ = 0.71073 Å
Hall symbol: -P 2yn	Cell parameters from 16004 reflections
*a* = 8.1184 (4) Å	θ = 1.9–27.2°
*b* = 22.9372 (13) Å	µ = 1.64 mm^−^^1^
*c* = 8.3417 (4) Å	*T* = 100 K
β = 116.443 (3)°	Block, light red
*V* = 1390.82 (12) Å^3^	0.24 × 0.15 × 0.09 mm
*Z* = 4	

### Data collection

**Table:**

Stoe IPDS 2T diffractometer	2935 independent reflections
Radiation source: fine-focus sealed tube	2753 reflections with *I* > 2σ(*I*)
graphite	*R*_int_ = 0.051
Detector resolution: 6.67 pixels mm^-1^	θ_max_ = 26.7°, θ_min_ = 2.9°
rotation method scans	*h* = −10→10
Absorption correction: multi-scan (Blessing, 1995)	*k* = −28→29
*T*_min_ = 0.630, *T*_max_ = 0.994	*l* = −9→10
9737 measured reflections	

### Refinement

**Table:**

Refinement on *F*^2^	Primary atom site location: structure-invariant direct methods
Least-squares matrix: full	Secondary atom site location: difference Fourier map
*R*[*F*^2^ > 2σ(*F*^2^)] = 0.017	Hydrogen site location: inferred from neighbouring sites
*wR*(*F*^2^) = 0.044	H-atom parameters constrained
*S* = 1.04	*w* = 1/[σ^2^(*F*_o_^2^) + (0.0208*P*)^2^ + 0.742*P*] where *P* = (*F*_o_^2^ + 2*F*_c_^2^)/3
2935 reflections	(Δ/σ)_max_ = 0.002
150 parameters	Δρ_max_ = 0.42 e Å^−^^3^
0 restraints	Δρ_min_ = −0.71 e Å^−^^3^

### Special details

**Table:**

Experimental. Anal. calc. for C_11_H_18_Cl_2_ORuS (370.29 g/mol) C 35.68, H 4.90%; found C 35.42, H 4.98%. ^1^H NMR (250 MHz, DMSO-*d*_6_, 300 K) δ = 2.14 (s, 9H, CH_3_), 2.54 (s, 6H, DMSO), 5.46 (s, 3H, CH) p.p.m.; ^13^C NMR (75 MHz, DMSO-*d*_6_, 300 K) δ = 18.2 (CH_3_), 40.4 (DMSO), 82.0 (CH), 104.8 (*C*CH_3_) p.p.m.. IR (neat, ATR) ν = 3063 w, 3024 m, 2963 w, 2931 w, 1525 m, 1447 m, 1408 m, 1377 m, 1303 m, 1286 m, 1105 s, 1034 m, 1008 *versus*, 983 s, 971 m, 932 m, 905 m, 887 m, 721 m, 680 m, 641 m, 507 w, 415 s cm^-1^.
Geometry. All s.u.'s (except the s.u. in the dihedral angle between two l.s. planes) are estimated using the full covariance matrix. The cell s.u.'s are taken into account individually in the estimation of s.u.'s in distances, angles and torsion angles; correlations between s.u.'s in cell parameters are only used when they are defined by crystal symmetry. An approximate (isotropic) treatment of cell s.u.'s is used for estimating s.u.'s involving l.s. planes.
Refinement. Refinement of *F*^2^ against ALL reflections. The weighted *R*-factor *wR* and goodness of fit *S* are based on *F*^2^, conventional *R*-factors *R* are based on *F*, with *F* set to zero for negative *F*^2^. The threshold expression of *F*^2^ > 2σ(*F*^2^) is used only for calculating *R*-factors(gt) *etc*. and is not relevant to the choice of reflections for refinement. *R*-factors based on *F*^2^ are statistically about twice as large as those based on *F*, and *R*- factors based on ALL data will be even larger.

### Fractional atomic coordinates and isotropic or equivalent isotropic displacement parameters (Å^2^)

**Table:**

	*x*	*y*	*z*	*U*_iso_*/*U*_eq_	
C1	0.1365 (2)	0.08700 (8)	−0.1389 (2)	0.0176 (4)	
C2	0.2204 (2)	0.03526 (7)	−0.0475 (2)	0.0172 (3)	
H2	0.2639	0.0074	−0.1039	0.021*	
C3	0.2406 (2)	0.02437 (7)	0.1282 (2)	0.0168 (3)	
C4	0.1758 (2)	0.06592 (7)	0.2106 (2)	0.0162 (3)	
H4	0.1889	0.0588	0.3279	0.019*	
C5	0.0910 (2)	0.11842 (7)	0.1211 (2)	0.0161 (3)	
C6	0.0741 (2)	0.12862 (7)	−0.0535 (2)	0.0168 (3)	
H6	0.0201	0.1639	−0.1137	0.020*	
C7	0.1190 (3)	0.09819 (10)	−0.3227 (2)	0.0283 (4)	
H7A	0.2138	0.0762	−0.3392	0.042*	
H7B	0.1348	0.1399	−0.3369	0.042*	
H7C	−0.0030	0.0858	−0.4122	0.042*	
C8	0.3318 (3)	−0.03050 (8)	0.2244 (3)	0.0257 (4)	
H8A	0.3877	−0.0238	0.3539	0.039*	
H8B	0.4273	−0.0420	0.1891	0.039*	
H8C	0.2400	−0.0616	0.1930	0.039*	
C9	0.0139 (2)	0.16108 (8)	0.2056 (3)	0.0244 (4)	
H9A	−0.1136	0.1506	0.1753	0.037*	
H9B	0.0174	0.2004	0.1609	0.037*	
H9C	0.0875	0.1602	0.3359	0.037*	
C10	0.5260 (2)	0.16583 (7)	0.5447 (2)	0.0178 (3)	
H10A	0.5907	0.1970	0.6298	0.027*	
H10B	0.6034	0.1309	0.5755	0.027*	
H10C	0.4105	0.1570	0.5500	0.027*	
C11	0.7007 (2)	0.21278 (7)	0.3628 (2)	0.0169 (3)	
H11A	0.6939	0.2315	0.2544	0.025*	
H11B	0.7841	0.1793	0.3935	0.025*	
H11C	0.7470	0.2408	0.4618	0.025*	
O1	0.35990 (15)	0.24130 (5)	0.28238 (15)	0.0164 (2)	
S1	0.47771 (5)	0.188752 (16)	0.32416 (5)	0.01120 (8)	
Cl1	0.65955 (5)	0.066716 (17)	0.30769 (5)	0.01704 (9)	
Cl2	0.50102 (5)	0.159381 (18)	−0.04032 (5)	0.01767 (9)	
Ru1	0.364774 (16)	0.110410 (5)	0.124893 (15)	0.01033 (5)	

### Atomic displacement parameters (Å^2^)

**Table:**

	*U*^11^	*U*^22^	*U*^33^	*U*^12^	*U*^13^	*U*^23^
C1	0.0100 (8)	0.0246 (9)	0.0132 (8)	−0.0059 (6)	0.0007 (6)	−0.0026 (6)
C2	0.0133 (8)	0.0158 (7)	0.0200 (8)	−0.0048 (6)	0.0052 (6)	−0.0089 (6)
C3	0.0126 (7)	0.0115 (7)	0.0229 (8)	−0.0046 (6)	0.0048 (6)	−0.0011 (6)
C4	0.0138 (8)	0.0175 (8)	0.0167 (8)	−0.0050 (6)	0.0062 (6)	0.0005 (6)
C5	0.0102 (8)	0.0152 (7)	0.0219 (8)	−0.0033 (6)	0.0063 (7)	−0.0044 (6)
C6	0.0075 (7)	0.0165 (8)	0.0200 (8)	−0.0007 (6)	0.0003 (6)	0.0032 (6)
C7	0.0219 (10)	0.0449 (11)	0.0126 (8)	−0.0066 (8)	0.0029 (7)	−0.0010 (8)
C8	0.0215 (9)	0.0144 (8)	0.0358 (10)	−0.0009 (7)	0.0079 (8)	0.0028 (7)
C9	0.0184 (9)	0.0226 (9)	0.0368 (10)	−0.0024 (7)	0.0165 (8)	−0.0075 (8)
C10	0.0231 (9)	0.0162 (8)	0.0126 (7)	−0.0017 (7)	0.0067 (7)	−0.0004 (6)
C11	0.0140 (8)	0.0169 (8)	0.0189 (8)	−0.0046 (6)	0.0065 (6)	−0.0047 (6)
O1	0.0164 (6)	0.0114 (5)	0.0179 (5)	0.0031 (4)	0.0044 (5)	−0.0005 (4)
S1	0.01161 (18)	0.00975 (17)	0.01084 (17)	−0.00026 (13)	0.00373 (14)	−0.00063 (13)
Cl1	0.01246 (19)	0.01451 (18)	0.01854 (19)	0.00243 (14)	0.00185 (15)	−0.00313 (14)
Cl2	0.01695 (19)	0.0227 (2)	0.01450 (18)	−0.00450 (15)	0.00802 (15)	−0.00160 (14)
Ru1	0.00945 (8)	0.00984 (7)	0.01029 (8)	−0.00034 (4)	0.00314 (5)	−0.00124 (4)

### Geometric parameters (Å, °)

**Table:**

C1—C2	1.411 (2)	C7—H7C	0.9800
C1—C6	1.413 (3)	C8—H8A	0.9800
C1—C7	1.498 (2)	C8—H8B	0.9800
C1—Ru1	2.2209 (15)	C8—H8C	0.9800
C2—C3	1.423 (2)	C9—H9A	0.9800
C2—Ru1	2.2162 (15)	C9—H9B	0.9800
C2—H2	0.9500	C9—H9C	0.9800
C3—C4	1.407 (2)	C10—S1	1.7807 (16)
C3—C8	1.499 (2)	C10—H10A	0.9800
C3—Ru1	2.2219 (16)	C10—H10B	0.9800
C4—C5	1.423 (2)	C10—H10C	0.9800
C4—Ru1	2.2097 (16)	C11—S1	1.7792 (17)
C4—H4	0.9500	C11—H11A	0.9800
C5—C6	1.420 (2)	C11—H11B	0.9800
C5—C9	1.496 (2)	C11—H11C	0.9800
C5—Ru1	2.2160 (17)	O1—S1	1.4806 (11)
C6—Ru1	2.1978 (15)	S1—Ru1	2.3399 (4)
C6—H6	0.9500	Cl1—Ru1	2.4097 (4)
C7—H7A	0.9800	Cl2—Ru1	2.3963 (4)
C7—H7B	0.9800		
			
C2—C1—C6	119.41 (15)	S1—C10—H10A	109.5
C2—C1—C7	120.01 (17)	S1—C10—H10B	109.5
C6—C1—C7	120.56 (17)	H10A—C10—H10B	109.5
C2—C1—Ru1	71.28 (9)	S1—C10—H10C	109.5
C6—C1—Ru1	70.47 (9)	H10A—C10—H10C	109.5
C7—C1—Ru1	129.03 (12)	H10B—C10—H10C	109.5
C1—C2—C3	120.71 (15)	S1—C11—H11A	109.5
C1—C2—Ru1	71.64 (9)	S1—C11—H11B	109.5
C3—C2—Ru1	71.52 (9)	H11A—C11—H11B	109.5
C1—C2—H2	119.6	S1—C11—H11C	109.5
C3—C2—H2	119.6	H11A—C11—H11C	109.5
Ru1—C2—H2	129.7	H11B—C11—H11C	109.5
C4—C3—C2	119.20 (15)	O1—S1—C11	106.80 (8)
C4—C3—C8	120.71 (16)	O1—S1—C10	107.79 (8)
C2—C3—C8	120.09 (16)	C11—S1—C10	99.56 (8)
C4—C3—Ru1	71.02 (9)	O1—S1—Ru1	116.76 (5)
C2—C3—Ru1	71.09 (9)	C11—S1—Ru1	114.32 (6)
C8—C3—Ru1	129.33 (12)	C10—S1—Ru1	110.09 (6)
C3—C4—C5	121.05 (16)	C6—Ru1—C4	67.42 (6)
C3—C4—Ru1	71.97 (10)	C6—Ru1—C5	37.53 (6)
C5—C4—Ru1	71.49 (10)	C4—Ru1—C5	37.50 (6)
C3—C4—H4	119.5	C6—Ru1—C2	67.06 (6)
C5—C4—H4	119.5	C4—Ru1—C2	66.92 (6)
Ru1—C4—H4	129.6	C5—Ru1—C2	79.51 (6)
C6—C5—C4	118.76 (15)	C6—Ru1—C1	37.29 (7)
C6—C5—C9	120.37 (16)	C4—Ru1—C1	79.38 (6)
C4—C5—C9	120.83 (16)	C5—Ru1—C1	67.47 (6)
C6—C5—Ru1	70.54 (9)	C2—Ru1—C1	37.08 (6)
C4—C5—Ru1	71.01 (9)	C6—Ru1—C3	79.68 (6)
C9—C5—Ru1	132.43 (12)	C4—Ru1—C3	37.01 (6)
C1—C6—C5	120.86 (15)	C5—Ru1—C3	67.42 (6)
C1—C6—Ru1	72.24 (9)	C2—Ru1—C3	37.40 (6)
C5—C6—Ru1	71.93 (9)	C1—Ru1—C3	67.33 (6)
C1—C6—H6	119.6	C6—Ru1—S1	107.34 (5)
C5—C6—H6	119.6	C4—Ru1—S1	103.51 (4)
Ru1—C6—H6	128.6	C5—Ru1—S1	91.13 (4)
C1—C7—H7A	109.5	C2—Ru1—S1	170.04 (5)
C1—C7—H7B	109.5	C1—Ru1—S1	141.22 (5)
H7A—C7—H7B	109.5	C3—Ru1—S1	135.20 (5)
C1—C7—H7C	109.5	C6—Ru1—Cl2	98.80 (5)
H7A—C7—H7C	109.5	C4—Ru1—Cl2	165.32 (4)
H7B—C7—H7C	109.5	C5—Ru1—Cl2	132.05 (5)
C3—C8—H8A	109.5	C2—Ru1—Cl2	103.74 (5)
C3—C8—H8B	109.5	C1—Ru1—Cl2	86.49 (5)
H8A—C8—H8B	109.5	C3—Ru1—Cl2	138.75 (5)
C3—C8—H8C	109.5	S1—Ru1—Cl2	85.030 (14)
H8A—C8—H8C	109.5	C6—Ru1—Cl1	166.37 (4)
H8B—C8—H8C	109.5	C4—Ru1—Cl1	103.89 (4)
C5—C9—H9A	109.5	C5—Ru1—Cl1	138.64 (5)
C5—C9—H9B	109.5	C2—Ru1—Cl1	100.16 (5)
H9A—C9—H9B	109.5	C1—Ru1—Cl1	132.98 (5)
C5—C9—H9C	109.5	C3—Ru1—Cl1	87.19 (4)
H9A—C9—H9C	109.5	S1—Ru1—Cl1	84.567 (14)
H9B—C9—H9C	109.5	Cl2—Ru1—Cl1	88.636 (15)
			
C6—C1—C2—C3	0.8 (2)	C9—C5—Ru1—Cl1	86.88 (18)
C7—C1—C2—C3	179.05 (15)	C1—C2—Ru1—C6	29.22 (10)
Ru1—C1—C2—C3	54.15 (14)	C3—C2—Ru1—C6	−103.49 (11)
C6—C1—C2—Ru1	−53.39 (13)	C1—C2—Ru1—C4	103.39 (11)
C7—C1—C2—Ru1	124.90 (15)	C3—C2—Ru1—C4	−29.32 (10)
C1—C2—C3—C4	0.0 (2)	C1—C2—Ru1—C5	66.34 (10)
Ru1—C2—C3—C4	54.18 (13)	C3—C2—Ru1—C5	−66.37 (10)
C1—C2—C3—C8	−179.36 (15)	C3—C2—Ru1—C1	−132.71 (15)
Ru1—C2—C3—C8	−125.15 (15)	C1—C2—Ru1—C3	132.71 (15)
C1—C2—C3—Ru1	−54.21 (14)	C1—C2—Ru1—Cl2	−64.65 (10)
C2—C3—C4—C5	−0.1 (2)	C3—C2—Ru1—Cl2	162.64 (9)
C8—C3—C4—C5	179.26 (15)	C1—C2—Ru1—Cl1	−155.73 (9)
Ru1—C3—C4—C5	54.15 (14)	C3—C2—Ru1—Cl1	71.55 (9)
C2—C3—C4—Ru1	−54.21 (13)	C2—C1—Ru1—C6	−132.10 (15)
C8—C3—C4—Ru1	125.12 (15)	C7—C1—Ru1—C6	114.0 (2)
C3—C4—C5—C6	−0.6 (2)	C2—C1—Ru1—C4	−65.58 (10)
Ru1—C4—C5—C6	53.79 (13)	C6—C1—Ru1—C4	66.52 (10)
C3—C4—C5—C9	176.92 (15)	C7—C1—Ru1—C4	−179.48 (19)
Ru1—C4—C5—C9	−128.72 (15)	C2—C1—Ru1—C5	−102.83 (11)
C3—C4—C5—Ru1	−54.37 (14)	C6—C1—Ru1—C5	29.27 (10)
C2—C1—C6—C5	−1.4 (2)	C7—C1—Ru1—C5	143.28 (19)
C7—C1—C6—C5	−179.70 (15)	C6—C1—Ru1—C2	132.10 (15)
Ru1—C1—C6—C5	−55.19 (13)	C7—C1—Ru1—C2	−113.9 (2)
C2—C1—C6—Ru1	53.77 (13)	C2—C1—Ru1—C3	−28.92 (10)
C7—C1—C6—Ru1	−124.51 (15)	C6—C1—Ru1—C3	103.18 (11)
C4—C5—C6—C1	1.3 (2)	C7—C1—Ru1—C3	−142.81 (19)
C9—C5—C6—C1	−176.18 (15)	C2—C1—Ru1—S1	−163.99 (8)
Ru1—C5—C6—C1	55.34 (13)	C6—C1—Ru1—S1	−31.89 (13)
C4—C5—C6—Ru1	−54.01 (13)	C7—C1—Ru1—S1	82.11 (19)
C9—C5—C6—Ru1	128.48 (15)	C2—C1—Ru1—Cl2	118.42 (10)
C1—C6—Ru1—C4	−102.51 (11)	C6—C1—Ru1—Cl2	−109.48 (9)
C5—C6—Ru1—C4	29.64 (9)	C7—C1—Ru1—Cl2	4.53 (17)
C1—C6—Ru1—C5	−132.15 (14)	C2—C1—Ru1—Cl1	33.57 (12)
C1—C6—Ru1—C2	−29.07 (10)	C6—C1—Ru1—Cl1	165.67 (8)
C5—C6—Ru1—C2	103.08 (11)	C7—C1—Ru1—Cl1	−80.32 (19)
C5—C6—Ru1—C1	132.15 (14)	C4—C3—Ru1—C6	−66.00 (10)
C1—C6—Ru1—C3	−65.95 (10)	C2—C3—Ru1—C6	65.54 (10)
C5—C6—Ru1—C3	66.20 (10)	C8—C3—Ru1—C6	179.40 (18)
C1—C6—Ru1—S1	159.72 (9)	C2—C3—Ru1—C4	131.54 (14)
C5—C6—Ru1—S1	−68.13 (9)	C8—C3—Ru1—C4	−114.6 (2)
C1—C6—Ru1—Cl2	72.21 (10)	C4—C3—Ru1—C5	−28.87 (9)
C5—C6—Ru1—Cl2	−155.64 (9)	C2—C3—Ru1—C5	102.67 (11)
C1—C6—Ru1—Cl1	−50.2 (2)	C8—C3—Ru1—C5	−143.47 (18)
C5—C6—Ru1—Cl1	81.9 (2)	C4—C3—Ru1—C2	−131.54 (14)
C3—C4—Ru1—C6	103.26 (11)	C8—C3—Ru1—C2	113.9 (2)
C5—C4—Ru1—C6	−29.67 (9)	C4—C3—Ru1—C1	−102.85 (11)
C3—C4—Ru1—C5	132.92 (14)	C2—C3—Ru1—C1	28.69 (10)
C3—C4—Ru1—C2	29.61 (9)	C8—C3—Ru1—C1	142.55 (18)
C5—C4—Ru1—C2	−103.31 (11)	C4—C3—Ru1—S1	38.27 (12)
C3—C4—Ru1—C1	66.25 (10)	C2—C3—Ru1—S1	169.81 (8)
C5—C4—Ru1—C1	−66.67 (10)	C8—C3—Ru1—S1	−76.33 (18)
C5—C4—Ru1—C3	−132.92 (14)	C4—C3—Ru1—Cl2	−157.62 (8)
C3—C4—Ru1—S1	−153.33 (9)	C2—C3—Ru1—Cl2	−26.08 (13)
C5—C4—Ru1—S1	73.75 (9)	C8—C3—Ru1—Cl2	87.78 (17)
C3—C4—Ru1—Cl2	82.2 (2)	C4—C3—Ru1—Cl1	117.67 (9)
C5—C4—Ru1—Cl2	−50.7 (2)	C2—C3—Ru1—Cl1	−110.79 (9)
C3—C4—Ru1—Cl1	−65.67 (9)	C8—C3—Ru1—Cl1	3.07 (16)
C5—C4—Ru1—Cl1	161.41 (8)	O1—S1—Ru1—C6	−11.73 (8)
C4—C5—Ru1—C6	131.39 (14)	C11—S1—Ru1—C6	−137.40 (8)
C9—C5—Ru1—C6	−113.8 (2)	C10—S1—Ru1—C6	111.55 (8)
C6—C5—Ru1—C4	−131.39 (14)	O1—S1—Ru1—C4	−81.94 (7)
C9—C5—Ru1—C4	114.8 (2)	C11—S1—Ru1—C4	152.39 (8)
C6—C5—Ru1—C2	−65.82 (10)	C10—S1—Ru1—C4	41.34 (8)
C4—C5—Ru1—C2	65.57 (10)	O1—S1—Ru1—C5	−46.16 (7)
C9—C5—Ru1—C2	−179.61 (18)	C11—S1—Ru1—C5	−171.83 (8)
C6—C5—Ru1—C1	−29.10 (10)	C10—S1—Ru1—C5	77.11 (8)
C4—C5—Ru1—C1	102.29 (11)	O1—S1—Ru1—C1	7.87 (9)
C9—C5—Ru1—C1	−142.89 (19)	C11—S1—Ru1—C1	−117.80 (9)
C6—C5—Ru1—C3	−102.88 (10)	C10—S1—Ru1—C1	131.14 (9)
C4—C5—Ru1—C3	28.51 (10)	O1—S1—Ru1—C3	−104.49 (8)
C9—C5—Ru1—C3	143.33 (19)	C11—S1—Ru1—C3	129.85 (9)
C6—C5—Ru1—S1	117.62 (9)	C10—S1—Ru1—C3	18.79 (9)
C4—C5—Ru1—S1	−110.99 (9)	O1—S1—Ru1—Cl2	85.96 (6)
C9—C5—Ru1—S1	3.83 (17)	C11—S1—Ru1—Cl2	−39.71 (6)
C6—C5—Ru1—Cl2	33.29 (11)	C10—S1—Ru1—Cl2	−150.77 (6)
C4—C5—Ru1—Cl2	164.69 (7)	O1—S1—Ru1—Cl1	175.05 (6)
C9—C5—Ru1—Cl2	−80.50 (18)	C11—S1—Ru1—Cl1	49.39 (6)
C6—C5—Ru1—Cl1	−159.32 (8)	C10—S1—Ru1—Cl1	−61.67 (6)
C4—C5—Ru1—Cl1	−27.93 (12)		
